# Preliminary Analysis of Osteocyte Lacunar Density in Long Bones of Tetrapods: All Measures Are Bigger in Sauropod Dinosaurs

**DOI:** 10.1371/journal.pone.0077109

**Published:** 2013-10-30

**Authors:** Koen W. H. Stein, Jan Werner

**Affiliations:** 1 Steinmann Institut für Geologie, Mineralogie und Paläontologie, University of Bonn, Bonn, Germany; 2 Institut für Zoologie, Abteilung Ökologie, Johannes Gutenberg-Universität Mainz, Mainz, Germany; Royal Ontario Museum, Canada

## Abstract

Osteocytes harbour much potential for paleobiological studies. Synchrotron radiation and spectroscopic analyses are providing fascinating data on osteocyte density, size and orientation in fossil taxa. However, such studies may be costly and time consuming. Here we describe an uncomplicated and inexpensive method to measure osteocyte lacunar densities in bone thin sections. We report on cell lacunar densities in the long bones of various extant and extinct tetrapods, with a focus on sauropodomorph dinosaurs, and how lacunar densities can help us understand bone formation rates in the iconic sauropod dinosaurs. Ordinary least square and phylogenetic generalized least square regressions suggest that sauropodomorphs have lacunar densities higher than scaled up or comparably sized mammals. We also found normal mammalian-like osteocyte densities for the extinct bovid *Myotragus*, questioning its crocodilian-like physiology. When accounting for body mass effects and phylogeny, growth rates are a main factor determining the density of the lacunocanalicular network. However, functional aspects most likely play an important role as well. Observed differences in cell strategies between mammals and dinosaurs likely illustrate the convergent nature of fast growing bone tissues in these groups.

## Introduction

### Osteocytes

Osteocytes and osteocyte characters observed in fossil bone provide an untapped reserve of information for paleobiological studies. Osteocyte features have recently been shown to provide information on growth rates as well as muscle attachment sites of extinct taxa [1], [2]. Moreover, osteocytes may have the potential to preserve proteins of extinct vertebrates [3], [4]. Osteocytes are the most common cells in intramembraneously formed bone tissues. They derive from bone forming osteoblasts which become incorporated into the bone matrix during bone growth (for a review see [5]). An osteocyte resides inside the bony tissue in an osteocyte lacuna, and remains in direct contact with other osteocytes through small pores called canaliculi [6]. This osteocyte canalicular network functions as a mechanosensing sensory network [7]–[9]. Osteocytes help maintain bone homeostasis by signalling other osteocytes, osteoblasts and osteoclasts about adjacent tissue damages or even changes in stress and strain in their local environment, inhibiting or promoting bone remodelling [8], [9], [10].

The factors determining the density of osteocytes in the lacunocanalicular network remain unclear. In comparison with teleost fishes, amphibians and other terrestrial vertebrates have much better developed osteocyte-lacunocanalicular systems, however, these differences may not be directly related to aquatic habitats [11]. Cubo et al. [1] found cellular density, among a number of other histomorphometric parameters, to be significantly correlated to femoral growth rate. Bromage et al. [12] found a relationship between the osteocyte density of lamellar bone and body mass of mammals. This relationship between osteocyte lacunar density (OLD) and body mass (BM) is described by an allometric function of the form OLD = a BM^b^ (or log OLD = log a+b log BM, to get a linear relationship). The exponent b has a negative value, indicating a decrease in OLD with increasing body mass. The authors concluded from this that OLD reflects the rate of osteoblast proliferation, transformation, and incorporation into bone as osteocytes during growth. Lacunar densities should therefore be higher in mammals with rapid growth, small body mass, and whose osteoblast proliferation rates would lead to higher osteocyte lacunar densities.

Osteocyte lacunar density may thus have the potential to provide significant information about bone cell proliferation, physiology and life history of vertebrates [1], [5], [12]. So far it has been the focus of (osteoporosis) studies in humans (e.g. [13]–[16]), and to some extent also in other mammals [17]–[21], [12]. Osteocytes themselves are rarely preserved in fossilized bone, but the lacunae provide a good proxy for the shape and maximum possible size as well as density of the osteocytes. Because lacunar density is a feature that can be measured relatively easily in fossil bone, it is surprising that almost no comparative data are known for extinct vertebrates.

### Sauropods and Bone Histology

Because of their unsurpassed body masses, sauropod dinosaurs have been the focus of an increasing number of paleohistological investigations [22]–[28]. Sauropod long bones are made of highly vascularized fast growing tissues, consisting of a thin woven bone trabecular framework compacted with highly organized primary bone (HOPB *sensu* [28]). In the diapsid lineage, these highly vascularized long bone tissues were already present in basal Archosauria [1], [29]–[31]. Highly vascularized long bone tissues can also be found in mammals, a feature which evolved basally in therapsids, possibly even in synapsids [32]–[33].

Life history features, like growth rates, are of high interest for many researchers studying bone histology (e.g. [34]–[42]. Most of these skeletochronological studies aim to model growth dynamics and estimate growth rates of tetrapods using lines of arrested growth (LAGs) or other types of growth mark (*cf.* [41]–[42]). In extant animals, LAGs and other growth marks are the result of a seasonal cessation or slowdown of growth respectively [43]–[46]. Skeletochronology, however has its limitations. In the femur of an alligator, Klein et al. [47] found a number of growth marks different from the actual known age. Sauropod dinosaurs only exceptionally preserve such growth marks in their long bones. In the case of the dwarfed sauropod *Europasaurus* [48], a tibia (DFMMh/FV495.5) and femur (DFMMh/FV495.9) of one individual show a different number of growth marks (6 and 4 respectively, KS, Pers. Obs.). Moreover, reported variabilities in the histology of different elements of other dinosaurs calls for caution in element selection and accounting for missing growth marks [49]. These complications make life history studies of sauropod dinosaurs difficult, and their growth rates not fully understood. Here we explore how paleocytological characters of sauropods and basal sauropodomorphs, compared to other tetrapods, can help us assess growth rates.

### Aim of the Study

High OLD indicates high cell proliferation rates and high local apposition and metabolic rates [1], [12]. Therefore, given the presence of highly vascularized bone tissues, high growth rates comparable to mammals [24], [50]–[52] and assumed high basal metabolic rates of sauropodomorphs (and dinosaurs in general), we hypothesized the OLD in sauropodomorphs to be similar to mammals. Furthermore, we hypothesize that OLD will decrease with body mass in Sauropodomorpha, because small taxa like *Saturnalia* should exhibit higher local apposition rates than large sauropods. Although overall increase in absolute body size in sauropods may be larger than in small sauropodomorphs, the local mitotic rates of the osteoblasts will be higher in these smaller taxa, similar to mammals. In a broader phylogenetic context, tetrapods with known low growth rates, like amphibians, crocodiles and squamate reptiles are hypothesized to have low OLD.

The aim of this preliminary investigation is thus to obtain a better understanding of the nature of the lacunocanalicular network in tetrapods, with a focus on sauropodomorphs, and following Stein and Prondvai [28] how sauropodomorph bone tissue is organized on a cellular level.

## Materials and Methods

We used thin sections of histological cores (cf. [25], [53] of long bones of 12 sauropodomorph taxa (*Saturnalia tupiniquim*, *Thecodontosaurus*, *Plateosaurus*, *Spinophorosaurus*, *Brachiosaurus*, *Europasaurus*, *Apatosaurus*, *Dicraeosaurus*, *Barosaurus*, *Janenschia*, *Phuwiangosaurus* and *Alamosaurus*, see Table 1) from the thin section collection at the Steinmann Institut in Bonn. Further sampled tetrapod taxa include a non-therapsid synapsid (*Dimetrodon natalis*, histological analysis in [54]), squamate reptiles (*Iguana iguana*, *Varanus niloticus*, *Varanus timorensis*, *Tupinambis teguixin*), basal archosauromorphs (*Trilophosaurus sp.*, *Hyperodapedon sp.*, *Rhamphorhynchus muensteri* (histological analysis in [39]), two alligators (*Alligator mississippiensis*, histological analysis in [47] and [55] respectively), large theropod dinosaurs (*Albertosaurus*, *Gorgosaurus* and *Tyrannosaurus*, histological analysis in [36], [6]–[57], and two birds (*Buteo buteo* and *Struthio camelus*). As non-amniote representatives, a Jurassic salamander (*Kokartus*, decribed in [58]), a common European frog (*Rana temporaria*) and *Diadectes sp.* were sampled. Mammal lacunar densities were taken from Bromage et al. [12] (Table 2). We measured OLDs of two additional extant mammal taxa, a guinea pig (*Cavia porcellus*) and an Indian elephant (*Elephas maximus*) to extend the range of body masses for mammals. Furthermore, we measured OLD in primary cortical bone of *Myotragus balearicus*, an extinct island-dwarf bovid, that has been reported to have a crocodile-like physiology and growth rate [59]. Thin sections of extant specimens were studied in their repository collections, or samples were taken from salvage specimens (i.e. animals which died of natural causes). All measured specimens with collection numbers, body mass estimates and osteocyte lacunar densities are listed in Table 1.

**Table 1:** Specimens with body masses and measured osteocyte densities.

Taxon	specimen nr.	et	el (mm)	BM (kg)	OLD (#/mm^3^)	BM source or method
***Kokartus***	ZiN.PH 43/47	fe		0.05	8601	P. Skutchas p.c.
***Rana temporaria***	IPB no nr.	fe		0.039	13828	species average
***Diadectes***	IPB no nr.	fe	130	35	29741	[99]
***Dimetrodon natalis***	IPB SABCBB 2010–26	fe	98	23	47413	[99]
***Dimetrodon natalis***	IPB SABCBB 2010–1	fe	108	28	34364	[99]
***Myotragus balearicus***	MBCN SM-T-8829-?-?	ti	183	20	26867	[100]
***Elephas maximus***	IPB no nr. female	fe		3000	19264	species average
***Cavia porcellus***	IPb no nr.	fe		0.7	36190	species average
***Iguana iguana***	AC 1896 288	fe	74.23	5	20534	V. de Buffrénil p.c.
***Tupinambis teguixin***	MK 53531/VB	fe		1.5	61118	V. de Buffrénil p.c.
***Varanus niloticus***	FAOTD39	fe		11	42977	V. de Buffrénil p.c.
***Varanus timorensis***	MK 52920	fe	33.41	0.8	53806	V. De Buffrénil p.c.
***Trilophosaurus***	TMM 31025-786	fe		14.8	37.037	[99]
***Trilophosaurus***	TMM 31025-885 avg.	fe		14.5	38117	[99]
***Trilophosaurus***	TMM 31025-67-02 avg.	fe		14.0	27051	[99]
***Trilophosaurus***	TMM 31025-67-01	fe		13.5	36.795	[99]
***Trilophosaurus***	TMM 31025-787	fe		13.3	27.505	[99]
***Hyperodapedon***	MCP PV0247	ti		188	23054	[99]
***Hyperodapedon***	MCP PV0407	hu		41	55787	[99]
***Hyperodapedon***	MCP PV408	hu		41	53129	[99]
***Rhamphorhynchus***	BSPG 1960 I 470a	ti		0.0834	52714	[39]
***Rhamphorhynchus***	BSPG 1929 I 69	fe		2.085	36859	[39]
***Rhamphorhynchus***	BSPG 1877 ×1	fe		0.112	46786	[39]
***Alligator mississippiensis***	SMNS 10481	fe		100	9064	[47]
***Alligator mississippiensis***	IPB “Babette” posterior	fe		6.86	18455	wet specimen measure
***Buteo buteo***	IPB no nr.	fe		1.3	59350	species average
***Struthio camelus***	IPB 5y old male	tt		115	46001	species average
***Gorgosaurus***	TMP 99.33.1	fi		607	15546	[36]
***Gorgosaurus***	TMP 99332	fi		607	17846	[36]
***Albertosaurus***	TMP 2002.45	fi		50.3	16294	[36]
***Albertosaurus***	TMP 86.64.1	fi		762	18790	[36]
***Albertosaurus***	TMP 86.64.1	fe		762	16765	[36]
***Albertosaurus***	TMP 81.10.1	fi		1142	17499	[36]
***Tyrannosaurus***	TMP 81.6.1	ilb		3230	13528	[36]
***Tyrannosaurus***	TMP 81.6.1	ilb		3230	12153	[36]
***Tyrannosaurus***	TMP 81.6.1	ilb		3230	12027	[36]
***Saturnalia***	MCP PV3845	fe		20	53432	[99]
***Thecodontosaurus***	IPB no nr.	ti		24.6	47611	[26]
***Plateosaurus***	SMNS F14A	fe	655	780	23300	[52]
***Plateosaurus***	SMNS F8	fe	740	900	20776	[52]
***Spinophorosaurus***	NMB 1698-R	hu	1121	6600	27392	U. Joger, p.c.
***Apatosaurus***	SMA “Jaques”	fe	1640	10000	33202	[26]
***Barosaurus***	MfN XVI5	fe	790	1500	45480	[99]
***Barosaurus***	MfN Ki2	fe	1190	11000	41878	[99]
***Dicraeosaurus***	MfN T31a	fe	980	3000	58540	[99]
***Dicraeosaurus***	MfN dd3032	fe	1140	4635	48500	[99]
***Europasaurus***	DFMMh/FV 415	fe	510	690	39386	[99]
***Brachiosaurus***	MfN dd452	fe	1350	10000	35647	[26]
***Brachiosaurus***	BYU 725-17336	fe	1750	19000	21923	[26]
***Janenschia***	MfN Nr.22	fe	1270	14029	43241	[26]
***Janenschia***	MfN Nr.22	fe	1270	14029	56715	[26]
***Phuwiangosaurus***	PC.DMR K21	fe	1120	9046	31866	[99]
***Alamosaurus***	TMM 43090-1	hu	1300	16000	26246	[99]

Abbreviations: **et**, element type (fe, femur; fi, fibula; hu, humerus; ilb, indeterminate long bone; ti, tibia; tt, tibiotarsus); **el**, element length (given where known); **BM**, body mass; **OLD**, osteocyte lacunar density; **p.c.**, personal communication. Institutional abbreviations: **BSPG**, Bayerische Staatssammlung für Paläontologie und Geologie; **BYU**, Earth Sciences Museum, Brigham Young University, Provo, Utah; **DFMMh/FV**, Dinosaurier-Freilichtmuseum Münchehagen/Verein zur Förderung der Niedersächsischen Paläontologie (e.V.), Germany; **IPB**, Institut für Paläontologie, Bonn, Germany; **MBCN**, Museu Balear de Ciències Naturals, Mallorca, Spain; **MCP,** Museu de Ciências e Tecnologia PUCRS, Porto Alegre, Brazil; **MFN**, Museum für Naturkunde; Berlin, Germany; **MK**, Museum König, Bonn, Germany; **NMB** Naturhistorisches Museum Braunschweig, Germany; **PC.DMR**, Paleontological Collection, Department of Mineral Resources, Khon Kaen Province, Kalasin, Thailand; **SMA**, Saurier Museum Aathal, Switzerland; **SMNS**, Staatliches Museum für Naturkunde Stuttgart, Germany; **TMM**, Texas Memorial Museum, Austin, Texas; **TMP**, Royal Tyrell Museum of Paleontology, Drumheller, Alberta, Canada. **ZiN.PH**, Zoological Institute, Russian Academy of Sciences, Paleoherpetological Collection, St. Petersburg, Russia.

**Table 2:** Mammal body masses and osteocyte lacunar densities from Bromage et[12].

Taxon	BM (kg)	OLD (#/mm^3^)
***Rattus norvegicus***	0.3	58000
***Phanourios minutus***	200	23641
***Hippopotamus amphibius***	2000	16667
***Otolemur crassicaudatus***	1.15	44353
***Chlorocebus aethiops***	3.515	32012
***Pan troglodytes***	33.7	18706
***Homo sapiens***	62	20444
***Galago moholi***	0.244	51724
***Cheirogales major***	0.4	31526
***Macaca mulatta***	3	22222

Bromage et al. [12] provided osteocyte densities of fully grown mammals. For a meaningful comparison, we required osteocyte densities of adult individuals. Therefore, we used only the largest individuals in our regression analyses if OLD’s of more than one individual of the same species were available. Additionally, osteocyte lacunar density was measured in the outer third of the bone cortex of the midshaft of transverse sections of mostly femora, but in some cases tibiae or humeri. In the case of sauropods, only individuals of at least histological ontogenetic stage 9 (HOS, [60]–[61]) were chosen. Sauropods of HOS 9 or above usually have laminar or plexiform bone with well defined primary osteons and a progressed state of cortical remodelling. Animals at this stage are not growing at the incredible juvenile rate anymore, and are putting more energy in maintenance than growth. Apart from being sexually mature, skeletal maturity may also have been reached if an EFS is present [60]. For all taxa with highly vascularized tissues, osteocyte lacunar density was measured in the parallel-fibred or rather highly organized primary bone (HOPB *sensu* [28]) matrix of the composite cortical bone. It should be noted that Hernandez et al. [19] found no significant difference between OLD of lamellar cortical bone and OLD of periosteal woven bone in the rat. However, Bromage et al. [12] measured lacunar density in HOPB, allowing direct comparison with their published data. The main reason for choosing HOPB to measure OLD is that taxa without highly vascularized long bone tissues only posess HOPB. Other reasons include the proportion of HOPB matrix is much larger than that of woven bone, which makes counting a significant number of osteocyte lacunae in woven bone nearly impossible; osteocyte lacunae in HOPB do not have irregular shapes as in woven bone, and are therefore easier to recognise with polarized light microscopic methods. Furthermore, a sample site without cracks, diagenetic alteration, and if possible, vascular canals, is also easier to locate.

**Figure 1:**
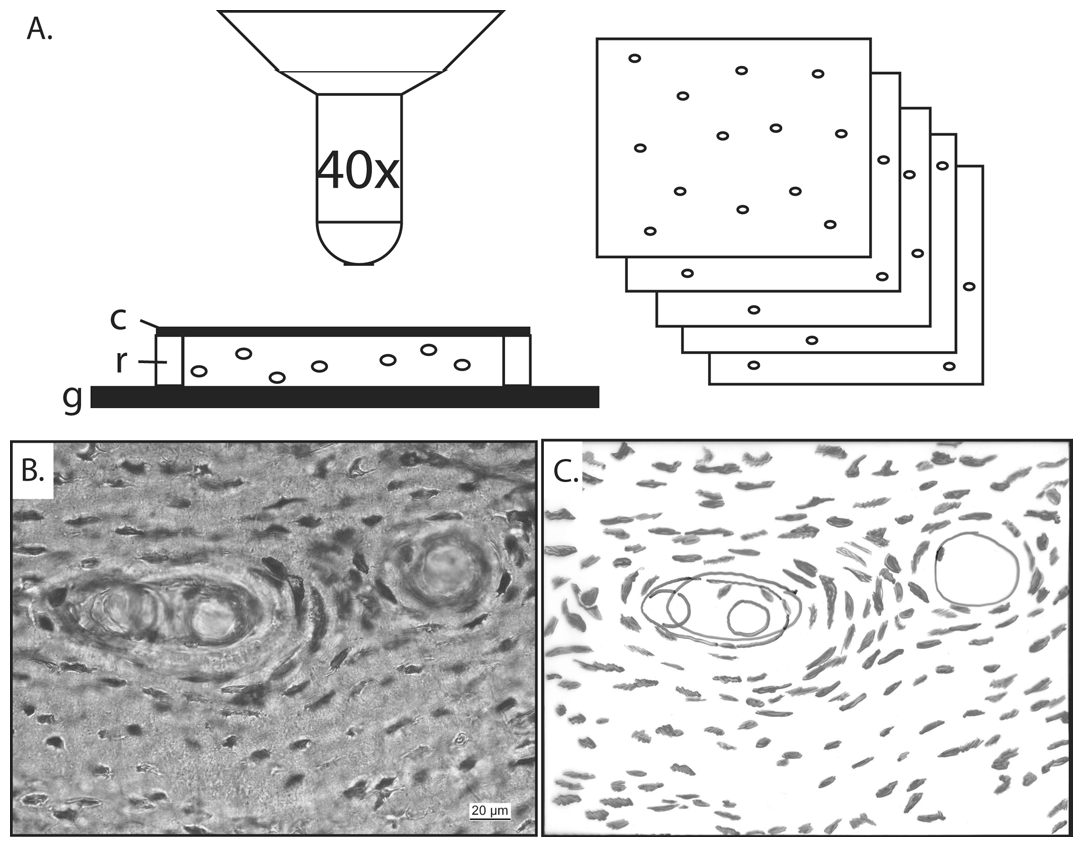
Method for acquiring z-stacks, and counting lacunae. A. Z-stack acquisition and thickness measurement. Thin sections were imaged at 40× magnification, the first image taken at the uppermost scratched surface of the specimen. The stage was then lowered with 5 µm for every subsequent image, until the lowermost scratched surface of the section was reached. Thickness of the sections was determined with a standard microscopic procedure. The sample was brought in focus on the upper surface of the epoxy resin. The stage was then lowered until the lower scratched surface of the epoxy resin was in focus. The difference in stage height setting, as read off the fine focus dial, was multiplied with the refractive index of the resin. This measurement was controlled with the number of images taken at 5 µm intervals. **B**,**C**, Lacunae identified in the z-stacks were projected on a two dimensional plane, and manually counted. The volume of bone was corrected for any vascular spaces, like in this example of *Dicraeosaurus*, any lacunae within the marked boundaries were ignored. The resulting volumetric density was then standardised to a volume of 1 mm^3^. **Abbreviations**: **c**, cover slip; **g**, glass slide; **r**, epoxy resin.

The method for measuring lacunar densities used here is similar to that of Bromage et al. [12], albeit with a less sophisticated, low-cost image processing technique (Fig. 1). Using a Leica DMLP microscope at 40× magnification, a 257-µm wide by 192 µm high XY field of view was chosen for each specimen as described above. Once an XY field was selected, a z-stack of images with a spacing of 5 µm was aquired using a Leica FireCam and processed with Leica Imageaccess software. Individual lacunae were identified in the three-dimensional image stacks and then projected on a two-dimensional plane. From these images, all identified lacunae were counted manually.

Thickness of the thin sections was determined with a standard microscopic procedure. The sample was brought in focus on the upper surface of the epoxy resin. The stage was then lowered until the lower scratched surface of the epoxy resin was in focus. The difference in stage height setting, as read off the fine focus dial, was multiplied with the refractive index of the resin. The Araldite™ two component resin used in our lab has a refractive index of 1.554 when hardened. This refractive index measure of the resin was provided by the manufacturer. The obtained thickness was controlled with the number of images in the z-stack with 5 µm distanced focal planes. The obtained thickness was multiplied with the surface area of the sample, corrected for any blood vessels, to obtain the total volume of bone. All measurements were then standardised by extrapolation of the number of lacunae per measured volume of bone to a 1 mm^3^ unit value.

To avoid the large potential errors involved in estimating body masses of extinct animals, femur length should be used as proxy for body size. However, longitudinal bone growth may be faster than appositonal growth in some species. Moreover, here, OLD is measured in a volume (i.e. 3 dimensions), and is thus more appropriately compared with a volumetric body mass. Bromage et al. [12] provided body masses obtained from literature species averages for their mammal samples (T. Bromage, Pers. Comm.). Body masses of the extinct animals in this study were collected from previously published literature sources or estimated with different methods (listed in Table 1). It should be emphasised that usage of sauropod dinosaur (but also other extinct animal) body mass estimates should be done with caution, as many well known potential problems are involved e.g. overestimating body density because of the airsac system, unknown humerus to femur ratio (see [62]–[63] for an introduction). However, the obtained body masses for this study were log-transformed to reduce this potential source of error.

Lacunar densities were log-transformed and plotted against the log-transformed body masses. Phylogenetic generalized least square regressions (PGLS, [64]–[66]) were calculated in R (version 2.15.2, [67]) for the whole dataset and separately for different groups containing at least six species (e.g. sauropodomorphs, mammals and reptiles, Table 3) using the generalized linear square method (gls) from the nlme package and the corPagel correlation structure from the ape package. Additionally we used a weighting structure in our PGLS analyses because the phylogenetic tree used was not ultrametric (containing extinct taxa). The weighting structure was calculated from the phylogeny by extracting the vector containing the branch lengths from the root to every tip (weights (W) = diag(vcv.phylo(tree)). Thus our final PGLS model was defined in R as

**Table 3:** Phylogenetic controlled regression models (log10 OLD = log10 intercept+slope * log10 BM) of osteocyte lacunar density (OLD) on body mass (BM) for different groups.

group	lambda	N	intercept	95% CI	SE	p-value	slope	95% CI	SE	p-value	AIC
All	0.987	42	4.530	[4.342, 4.719]	0.096	<0.001	−0.096	[−0.146, −0.047]	0.025	<0.001	−15.377
Mammals	0.552	13	4.584	[4.491, 4.676]	0.047	<0.001	−0.108	[−0.154, −0.063]	0.023	<0.001	−7.270
Reptiles	1.175	7	4.694	[4.329, 5.060]	0.186	<0.001	−0.159	[−0.338, 0.019]	0.091	0.141	9.565
Reptiles without *Alligator*	−0.592	6	4.677	[4.644, 4.710]	0.017	<0.001	−0.131	[−0.203, −0.058]	0.037	0.024	6.547
Sauropodomorphs	1.015	12	4.863	[4.627, 5.098]	0.120	<0.001	−0.130	[−0.234, −0.025]	0.053	0.036	−0.460

All = overall regression analyses with all available data. lambda = Pagel’s lambda. N = sample size. 95% CI = 95% confidence interval of the respective regression coefficient. SE = standard error. AIC = Akaike information criterion. For details on calculating the regression models see text.



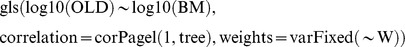
where OLD = osteocyte lacuna density, BM = body mass tree = phylogenetic tree, W = weights.

To solve the problem that no complete phylogeny was available for all species, we constructed a new tree based on different published phylogenetic trees. Branch lengths were calculated from estimated divergence times of the different nodes taken from the literature, because characters and clustering methods used to construct trees might have been different and thus might have affected branch lengths. Phylogenetic trees were constructed from Vidal and Hedges [68], Mulcahy et al. [69] and Amer and Kumazawa [70] for squamates; Hackett et al. [71] for birds; Marjanovic and Laurin [72] and Clack [73] for non-amniote tetrapods; Bennett [74] and Nesbitt [75] for non-dinosaurian archosaurs; Pisani et al. [76] and Brusatte et al. [77] for tyrannosaurid dinosaurs; Yates [78]–[79]; Sereno [80]; Allain and Aquesbi [81]; Remes et al. [82] for Sauropodomorpha and Beck et al. [83] and Perelman et al. [84] for mammals using Mesquite v. 2.75 [80]. Additional information on node divergence times was taken from Benton et al. [85] and Müller and Reisz [86]. Specific taxon ranges were obtained from the paleobiology database on 26/06/2013, except for *Phanourios minutus* ( = *Hippopotamus minutus*) and *Spinophorosaurus* for which stratigraphic data were obtained from Van der Geer et al. [87] and Remes et al. [82] respectively. A nexus file containing our calibrated tree can be found in Nexus S1.

To test for differences in slopes and in intercepts of the regressions of the different taxonomic groups we performed pairwise comparisons using t-tests. If variances of intercept or slope were statistically unequal t-test for unequal variances were used otherwise not. For further comparisons we also calculated the 95% prediction interval of the phylogenetic controlled mammal regression using standard methods.

## Results

**Figure 2:**
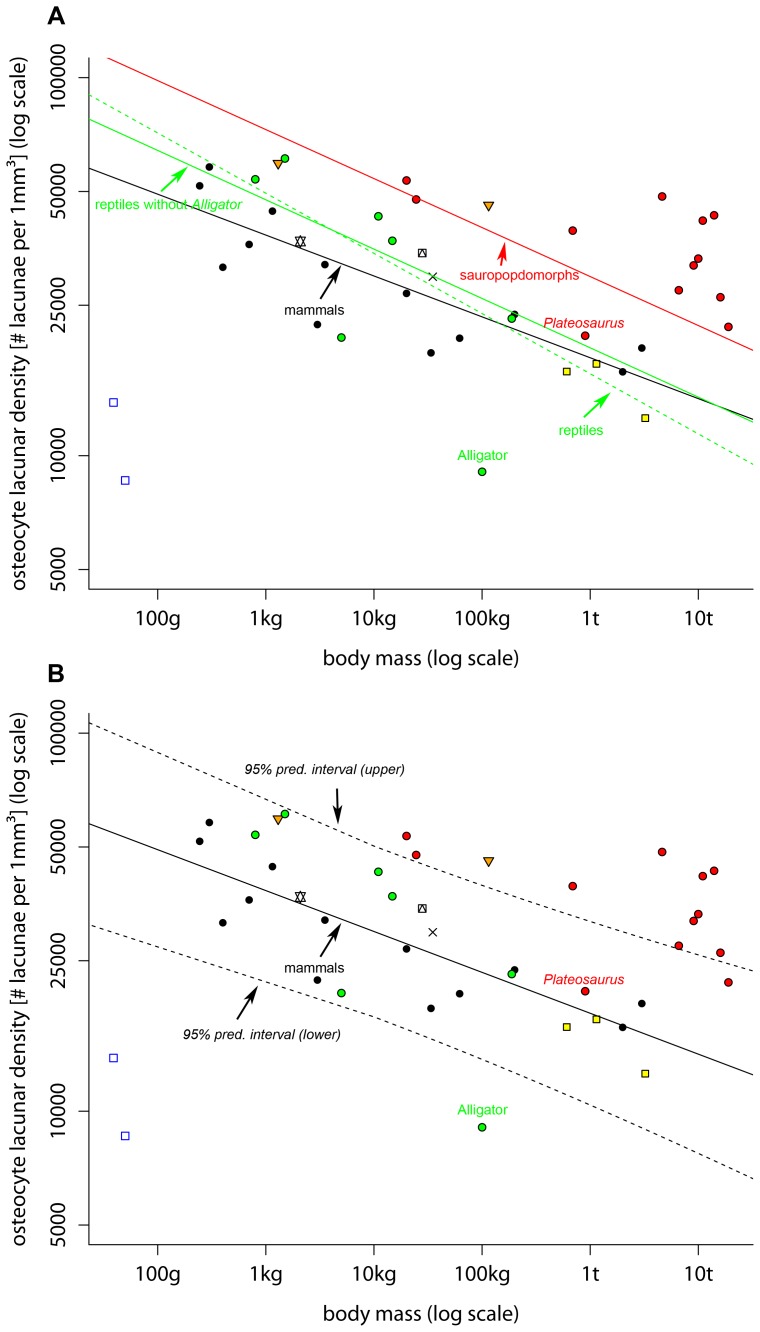
Visualisation of osteocyte lacuna densities in different tetrapods. A. Plot of osteocyte lacunar density on body mass of different taxa on a double logarithmic scale. Lines are the phylogenetic controlled regression lines of the respective taxonomic group. Solid lines represent significant regression models. The scattered line represents a regression model where the slope is not significant different from zero using a significant level of 0.05. For details of the regression models see Table 3. black circles  =  mammals, red circles  =  sauropodomorphs, blue open squares  =  amphibians, yellow squares  =  theropods, orange triangles  =  birds, green circles  =  reptiles, cross  =  diadectomorphs, star/pentagram  =  pterosaurs, square with triangle  =  “pelycosaurs”. **B**. Studied species in comparison to the mammal regression model (solid line). Scattered lines are 95% prediction intervals of the mammal regression model. Symbols as in **A**.

In general, OLD and BM were correlated with each other and this yielded to significant regression models except for reptiles (Table 3). With the exception of the regression model over all available data points and the reptile regression residuals were normally distributed (Shapiro-Wilk normality test; all: W = 0.9296, p-value = 0.01257; reptiles: W = 0.7924, p-value = 0.03442; mammals: W = 0.9592, p-value = 0.7407; sauropodomorphs: W = 0.9485, p-value = 0.6145) However, removing the *Alligator* from the reptile sample which might be an outlier (see Figure 2) produced a significant regression model for reptiles (Table 3) with normally distributed residuals (Shapiro-Wilk normality test; W = 0.8141, p-value = 0.07835). Comparing pairwise the taxonomic groups with each other, which contained at least six species (mammals, reptiles, reptiles without *Alligator*, sauropodomorphs), showed that all slopes were statistical not different (Table 4). Comparing the intercepts revealed that the sauropodomorph intercept was different from that of mammals and reptiles, whereas the intercepts of mammals and reptiles were statistical not different from each other (Table 4). However, the reptile regression model without the *Alligator* had a significant different intercept in comparison to the mammal regression. (Table 4).

**Table 4:** Pairwise comparisons of the slopes and intercepts of the different regression models (sauropodomorphs, mammals, reptiles, reptiles without *Alligator*). t = t-value of t-test, df = degree of freedom, p = p-value.

	Mammals		Reptiles		Reptiles without *Alligator*	
	intercepts	slopes	Intercepts	slopes	intercepts	slopes
Sauropodomorphs	1t = 7.519	1t = 1.283	t = 2.405	t = 0.901	1t = 5.242	t = 0.038
	df = 11	df = 11	df = 17	df = 17	df = 5	df = 16
	p<0.001	p = 0.226	p = 0.028	p = 0.380	p = 0.003	p = 0.960
Mammals			1t = 1.546	1t = 1.457	1t = 6.317	t = 1.619
			df = 6	df = 6	df = 5	df = 17
			p = 0.173	p = 0.195	p = 0.002	p = 0.124

1 t-test for unequal variances.

Using the mammal regression and the 95% prediction interval of the mammal regression as a baseline for comparison revealed that amphibians and the *Alligator* had low OLD’s in comparison to all other taxa (Figure 2B). The bird species *Struthio camelus*, and sauropodomorphs (with exception of *Plateosaurus* and) had higher OLD values as observed in mammals (Figure 2B). All other OLD’s were within the mammalian range considering so diverse taxa like theropods, extant ectothermic reptiles, reptiliomorphs, synapsids as well as Pterosauria (Figure 2B). A pairwise comparison [88] in Mesquite [89] yielded similar results, i.e. that there is a statistically significant relation between body mass and OLD (p = 0.039) (see Nexus S1.).

## Discussion

Our results suggest the relation between OLD and body mass is complex in nature. Carter et al. [90] found highly variable OLD within one single femur section of a young male human. Differences in osteocyte density up to 30%, combined with differences in general osteocyte morphology, between anterior, posterior, lateral and medial sides were strongly attributed to differences in mechanical loading regimes. Sanchez et al. [2], in their figure 4C, provide a visual representation of such variation in a virtual thin section of the humerus of the salamander *Desmognathus*. We also found strong lacunar density variation in a smaller specimen (femur TMM31025-885) of *Trilophosaurus sp.* (42601 lacunae/mm^3^ on the anterior side and 33632 lacunae/mm^3^ on the posterior side). Being aware of these variations, we tried to use standardized locations for measurements, with a sampling location midshaft on the anterior side of the femur. This requirement could not always be met. For example, Bromage et al. [12] did not specify precise locations of measurements, but also when dealing with fossil specimens a femur may not always be available, or preservational reasons prohibit sampling of the desired location. Moreover, a systematic approach with standardized sampling locations would ultimately also account for widely varying locomotion styles and resulting principal loading regimes in the sampled element. Nonetheless, in a general trend among mammals, dinosaurs and reptiles, OLD decreases with increasing body mass. Mullender et al. [18] found a similar relationship for the osteocyte lacunar densities within the cancellous bone tissues of the proximal femur in five mammals. Skedros et al. [91] also observed decreasing OLD with body mass in the turkey ulna. Moreover, they found high lacunar densities in the turkey compared to mammals of a similar size. High lacunar densities in the turkey ulna is consistent with works by Marotti et al. [92] and Remaggi et al. [93] who found high lacunar densities in the domestic chicken. Unfortunately, these authors used surface area measurements, making a direct comparison of the actual values with those presented here difficult. Nevertheless, the two bird species in our study (*Buteo buteo*, *Struthio camelus*) had high lacunar densities in comparison to mammals, too. Skedros et al. [91] speculated that substantially greater lacunar densities in avian species compared to mammals may be a function of their relatively higher specific metabolic rate (metabolic rate per kilogram of body mass), but did not provide further details.

Sauropods have unexpectedly high OLD-values, more than twice as high as expected for scaled up mammals. Also remarkable are the high OLD of the *Tupinambis* and monitor lizards. Even though the large alligator has much lower OLD compared to similar-sized mammals, the squamate high OLD are in contrast with the notion that OLD is directly related to basal metabolic rates. Concomitantly, the much higher lacunar density of the extinct insular bovid *Myotragus* compared to a similar-sized alligator would certainly question its presumed crocodilian metabolic physiology [59].

**Figure 3:**
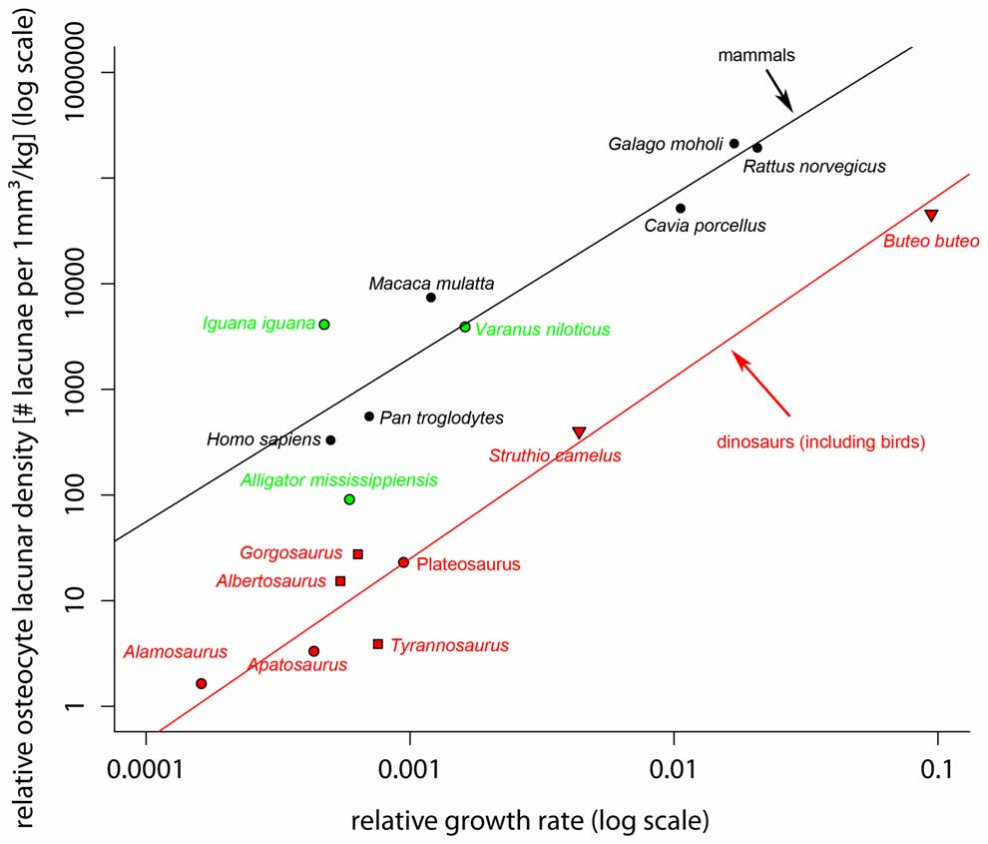
Plot of relative osteocyte lacunar density (ROLD, osteocyte lacunar density per 1 mm^3^/kg body mass) on relative growth rate (RGR, relative growth per day) on a double logarithmic scale. Red  =  dinosaurs including birds, black  =  mammals; green  =  reptiles. Solid lines are phylogenetic controlled regression models of the respective groups (dinosaurs including birds, mammals). Phylogenetic controlled regression models: log10 ROLD = 6.550 [5.897, 7.203]+1.718 [1.477, 1.958]*log10 RGR, AIC = 13.456, p<0.001, lambda = −0.420 (dinosaurs including birds); log10 ROLD = 7.939 [7.325, 8.553]+1.548 [1.548 1.548]*log10 RGR, AIC = 10.359, p<0.001, lambda = 1.111 (mammals). 95% confidence intervals of regression coefficients in square brackets. Residuals of both regressions were normally distributed (Shapiro-Wilk normality test; dinosaurs including birds: W = 0.955, p-value = 0.760; mammals: W = 0.900, p-value = 0.374). Regression lines were significant different from each other (t-test; slopes: t value = 3.917 df = 5, p = 0.011 intercepts: t value = 7.917, df = 12, p<0.001). Note: For the species *Rattus norvegicus* and the *Galago moholi* no RGR were available, therefore we used the RGR’s of phylogenetic closely related species (same genus) with similar body masses (*Rattus rattus*, *Galago senegalensis*).

In an attempt to further test the relation between osteocyte lacunar density and growth rate, we plotted OLD’s per kg body mass versus relative growth rates (RGR) for the taxa for which data were available (Figure 3). RGR’s are from Werner and Griebeler (this collection) and were calculated from fitted growth models as described in Fitzhugh [94] that is maximal growth rate (of the respective growth model) divided by the body mass at which this rate occurs. OLD’s were divided by body mass of the studied taxa to get mass-specific values, too. This approach should also account for body size scaling effects. Interestingly, OLD per kg body mass is significantly correlated with relative growth rate in dinosaurs (including birds) as well as in mammals. On a log-log plot, linear regression analyses show that the regression model for all dinosaurs (including birds) is significantly different from that for mammals (Figure 3). This means for a given lacunar density, dinosaurs (including birds) have a higher relative growth rate than mammals and probably also reptiles (Figure 3). The alligator was here taken together with reptiles because of similar physiologies, however, because the sample plots in between regression lines for mammals+reptiles and dinosaurs, it can arguably be taken together with dinosaurs, extending the dinosaur regression to crown-group archosaurs. This may be tested in future projects with other crurotarsal archosaurs with known relative growth rates.

Cao et al. [11] found much more developped lacunocanalicular networks in terrestrial tetrapods versus teleost fish. They suggested these differences may not be related to the aquatic habitat, however, the amphibians in our analysis, as well as the amphibious alligator have the lowest OLD values. The non-amniote *Diadectes* has relatively high OLD, but this animal probably was a relatively terrestrial animal [95]. Similarly, the actively hunting and foraging squamate reptiles also have high lacunar densities compared to the amphibious poikilotherm alligator. Locomotion and biomechanics thus most likely have a significant influence on the density of the lacunocanalicular network. Moreover, it is interesting to note that the bipedal *Plateosaurus* in our analysis have lacunar density values closer to those of theropods than to sauropods but not *Thecodontosaurus* and *Saturnalia*.

Other aspects of the lacunocanalicular network in tetrapod bones may reflect functional signals too. Rensberger and Watabe [96] observed differences between lacunocanalicular features in secondary osteons of theropod and birds and those of ornithopods and mammals. These features most likely do not represent true differences in lacunocanalicular morphology, but rather differences in the orientation of the osteocytes [28]. Nevertheless, the suggestion that birds and theropods have osteocytes oriented mostly parallel with the long bone axis, whereas ornithopods and mammals have osteocytes generally oriented more perpendicular to the long bone axis, may reflect differences in biomechanics and/or locomotion style. This hypothesis receives strong support from modern *in vivo* studies on bioapatite c-axis orientation [97], [98]. The hypotheses presented here can be tested by sampling large and small ornithischian dinosaurs, as well as a wider variety of theropods and birds, but also amphibians and squamate reptiles. To test the individual contributing effects of growth rates, principal mechanical loading and bone apposition rates on the density of the lacunocanalicular network, more detailed measurements of these features in vivo and analysis with variation partitioning methods are required. These are, however, beyond the scope of the current paper.

## Conclusions

The precise cause and origin of high lacunar densities in Sauropodomorpha relative to other tetrapods remains unclear to this point. Further testing on extant amniotes with known behavioural ecology, growth rate and metabolic rate may provide a better resolution on the factors determining osteocyte lacunar density. When accounting for body mass effects and phylogeny, growth rates are a main factor determining the density of the lacunocanalicular network. However, functional aspects most likely play an equally important determining role as well.

## Supporting Information

Nexus S1 Nexus file containing calibrated tree and analysis of osteocyte lacuna density with pairwise comparison.
(NEX)
